# Supplementary material for: Integrated analysis of the aqueous humor microbiome and lens capsule transcriptome in high myopia cataract: a pilot study
Source: Front Med (Lausanne). 2026 Jun 16;13:1845205. doi: 10.3389/fmed.2026.1845205 (PMC13314463; doi:10.3389/fmed.2026.1845205)
Supplement: Supplementary file 3 [file Data_Sheet_1.zip › 3.Beta_Diversity/Distance/C372089/bray_curtis_distance.pdf]

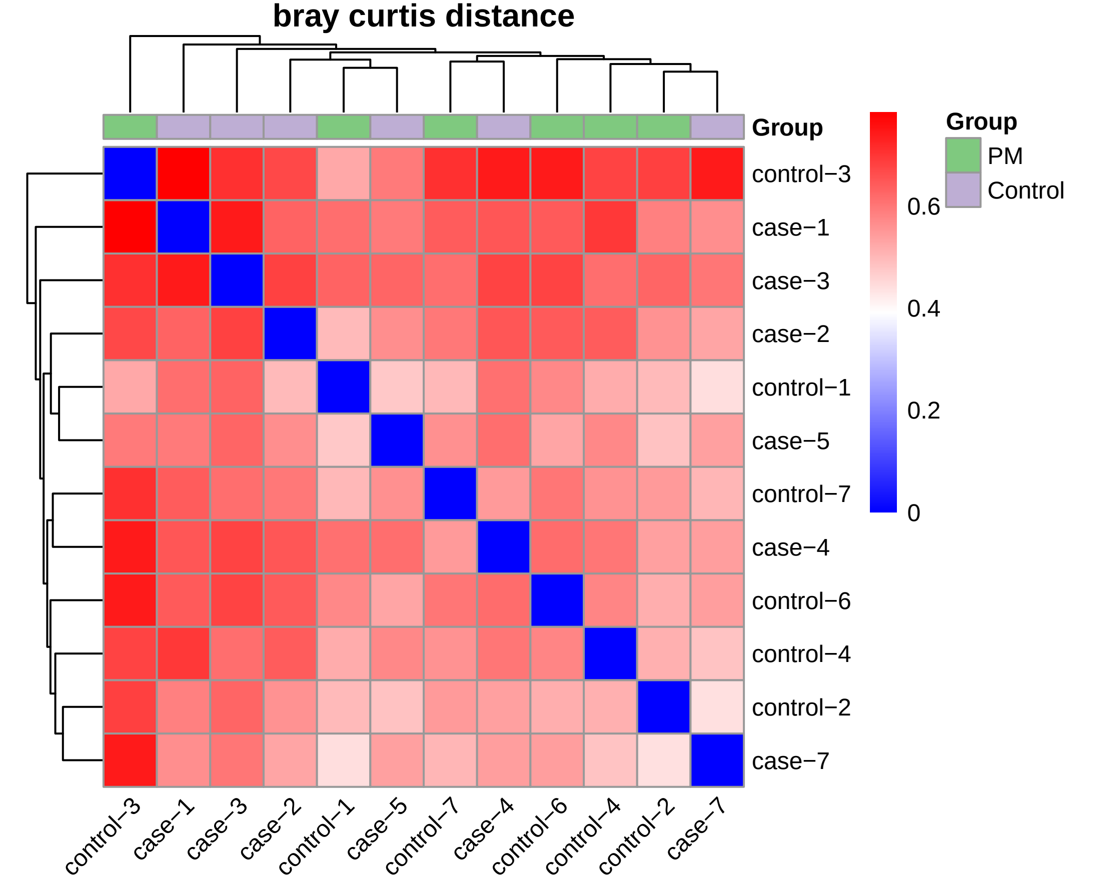

bray curtis distance
Group
control−3
case−1
case−3
case−2
control−1
case−5
control−7
case−4
control−6
control−4
control−2
case−7
0.6
0.4
0.2
0
Group
PM
Control
